# The use of tacit and explicit knowledge in public health: a qualitative study

**DOI:** 10.1186/1748-5908-7-20

**Published:** 2012-03-20

**Authors:** Anita Kothari, Debbie Rudman, Maureen Dobbins, Michael Rouse, Shannon Sibbald, Nancy Edwards

**Affiliations:** 1 Faculty of Health Sciences, The University of Western Ontario, Richmond Street, London, Canada; 2 School of Nursing, McMaster University, Main Street West, Hamilton, Canada; 3 Richard Ivey School of Business, The University of Western Ontario, Richmond Street, London, Canada; 4 Faculty of Health Sciences and Faculty of Medicine, University of Ottawa, Laurier Avenue East, Ottawa, Canada

**Keywords:** Knowledge translation, Tacit knowledge, Narrative inquiry, Public health, Program planning

## Abstract

**Background:**

Planning a public health initiative is both a science and an art. Public health practitioners work in a complex, often time-constrained environment, where formal research literature can be unavailable or uncertain. Consequently, public health practitioners often draw upon other forms of knowledge.

**Methods:**

Through use of one-on-one interviews and focus groups, we aimed to gain a better understanding of how tacit knowledge is used to inform program initiatives in public health. This study was designed as a narrative inquiry, which is based on the assumption that we make sense of the world by telling stories. Four public health units were purposively selected for maximum variation, based on geography and academic affiliation.

**Results:**

Analysis revealed different ways in which tacit knowledge was used to plan the public health program or initiative, including discovering the opportunity, bringing a team together, and working out program details (such as partnering, funding).

**Conclusions:**

The findings of this study demonstrate that tacit knowledge is drawn upon, and embedded within, various stages of the process of program planning in public health. The results will be useful in guiding the development of future knowledge translation strategies for public health organizations and decision makers.

## Background

The front-line public health system is vital for supporting disease prevention, health promotion, and healthy public policy initiatives. Over the past decade, a number of new and persistent health risks, such as resurgent infectious diseases (*e.g*., H1N1 influenza), threats of bioterrorism (*e.g*., anthrax), large-scale natural disasters (*e.g*., New Orleans, Haiti), and the advance of chronic diseases (*e.g*., diabetes, heart disease) have threatened the general population. One response has been a strengthened commitment to develop the science in public health knowledge exchange and uptake [1], also known as knowledge translation (KT). We take the position that KT is an interactive process of knowledge exchange between health researchers and health practitioners [2], or communities of practitioners [3]. In this paper, we focus on the tacit knowledge that is exchanged in a public health team.

Public health represents a unique setting deserving special attention with respect to KT. Public health practitioners must operate in a complex environment, often dealing with emergencies and disaster response, where the formal research literature related to practice is unavailable or uncertain. In addition, Kiefer *et al. *[1] suggest that there is limited capacity and skills among public health practitioners and decision-makers in the KT research-to-action process. Knowledge gained through years of experience in a local context is used, therefore, to augment or adapt the available research findings so that they are relevant and applicable for the local setting. As Landry *et al. *[4] point out, these local expert resources are currently undervalued.

Fundamental to this discussion is the distinction between explicit (codified, written) knowledge, typically represented by scientific research literature, and tacit ('know-how') knowledge, thought to be accumulated through previous knowledge, experience in local communities, and professional expertise [5-8]. We raise this distinction because a systematic exploration of tacit knowledge is markedly missing from the KT and public health literatures. That is, the literature on KT in public health has typically adopted a narrow scope, with formal, explicit scientific research represented as the knowledge or evidence being translated for use in practice and/or policy decision-making [6,9]. A notable, recent exception is found in the work of Landry *et al. *[4], who draw from the organizational management literature to develop a conceptual framework for KT in public health. Their knowledge-value chain (where knowledge is defined as the capacity to act) is a non-linear framework that outlines five capabilities necessary to manage knowledge, including mapping acquisition, creation and destruction, integration and sharing/transfer, replication and protection, and performance innovation. Tacit knowledge is discussed as an integral aspect of these capabilities. Thus, in line with the knowledge-value chain, we take a broad view of KT by encompassing other forms of knowledge that are experiential and skills-based (*i.e*., tacit). The specific purpose of this paper is to report findings from a narrative inquiry that examined how public health practitioners used tacit knowledge in a program planning context.

### Literature related to tacit knowledge

The term 'tacit' knowledge was first described by Polanyi, who stated, '...we can know more than we can tell' [10]. He described tacit knowledge as difficult to communicate and acquired through practice and experience rather than through language. According to Polanyi's concept, tacit knowledge is related to individual skills while embedded in context. Further, he saw tacit knowledge as inseparable from explicit knowledge. Terms like skills, intuition, know-how, procedural knowledge, implicit knowledge, unarticulated knowledge, and practical or experiential knowledge have all been used to describe tacit knowledge [11]. These terms reflect the fact that tacit knowledge has been conceptualized differently by various disciplines. Generally speaking, the empirical literature dealing with tacit knowledge in KT, and in public health, is sparse [11], representing an area to which the findings from this study will contribute.

Researchers highlight the notion that tacit knowledge is multidimensional and context-specific, and while it is often embedded within organizational routines, it is highly practice-related [9,11-13]. Descriptions of tacit knowledge, or 'know-how,' are sometimes presented in contrast to explicit, codified knowledge [14]. Other scholars take on Polanyi's view by rejecting an explicit/tacit dichotomy in favour of describing a continuum of knowledge [13]. There is no agreed upon definition of tacit knowledge, therefore we used a working definition from a review of the literature by McAdam *et al. *[15], who concluded that tacit knowledge is 'knowledge-in-practice developed from direct experience and action; highly pragmatic and situation specific; subconsciously understood and applied; difficult to articulate; usually shared through interactive conversation and shared experience.'

The organizational management literature positions tacit knowledge as a valuable resource that may be key to an organization's innovation and competitive advantage [13]. Several papers highlight how tacit knowledge is created [14], stored [16], and shared or exchanged [17]. Nonaka *et al. *describe a knowledge cycle model that can be used to conceptualize the place of tacit knowledge vis-à-vis explicit knowledge. Their model is broken down into four categories or modes of knowledge: externalization, combination, internalization, and socialization [14,18]. Externalization involves converting tacit knowledge into explicit knowledge; the process of combination entails creating new explicit knowledge from existing explicit knowledge; internalization is a process in which explicit knowledge is made tacit; finally, socialization involves the creation of tacit knowledge through shared experience. Individuals in an organization engage in these various modes of knowledge creation as they interact with one another, demonstrating that Nonaka and Toyama's model reflects a dialectical and dynamic process. Unlike Polanyi, Nonaka and Toyama suggest that a degree of tacit knowledge can be articulated [18].

Turning to health practitioners, there is a growing awareness of the different preferences for various types of knowledge in particular contexts. For example, Estabrooks *et al. *[19] found that nurses relied on social interactions, experience, documents, and *a priori *knowledge. They and other researchers [20] found that nurses frequently prefer experiential and interactive knowledge over more traditional formal sources (*i.e*., books, journals). Rycroft-Malone *et al. *[21] developed a general taxonomy of knowledge sources including research, professional knowledge/clinical practice, local information, and patient experiences/preferences; these authors challenge researchers to address the full range of knowledge sources that are used in clinical decision-making.

In the area of public health program planning, the emphasis still relies heavily on using codified knowledge to design and evaluate programs or services. This is in keeping with the popularity of the evidence-based practice movement, which values the use of explicit, scientific evidence in decision-making processes. Program planning frameworks and models abound, which Brownson *et al. *[22] define as a key tenet of evidence-based public health, noting that frameworks are often based on behavioural theories. Although many different frameworks exist, they tend to share the characteristics of being multistep and cyclical in nature. Steps involve a process of identifying a problem, defining goals and objectives, developing strategies to address the problem, implementing the strategies, and then evaluating the program through process and/or outcome indicators [23-28]. Examples of popular models include PRECEDE-PROCEED, logic models, and intervention mapping [29-31].

Within these frameworks are guidelines on different types of knowledge and evidence that should be used to inform decision-making throughout the planning process. Our review of public health planning models found that the emphasis in most models is on explicit knowledge. Sources of knowledge suggested include literature reviews, surveillance data, statistics from computerized patient records, key informant surveys, community forums and surveys, focus groups, mandates/guidelines for policies, and evaluations from other public health programs [23-25,28,32,33]. These sources of evidence can be used for multiple steps in the planning process, such as the identification of a problem, identifying potential strategies to address the problem, deciding upon a particular strategy, and evaluating the program. Tacit knowledge, on the other hand, is not referred to as a legitimate source of knowledge in planning frameworks, although there are opportunities for tacit knowledge to be used. Such opportunities could include determining stakeholder involvement and decision-making power, establishing program timing, determining resources available, assessing political agendas of others and the general political environment, assessing community readiness, and taking into account the local context [23,25-27]. Although planning frameworks identify these factors as important to consider, they do not provide clear guidance on how public health professionals should make these assessments, and make no suggestions on how to elicit tacit knowledge. The models that come closest to acknowledging tacit knowledge are the University of Toronto's The Health Communication Unit (TCHU) Planning Model, which refers to previous experience as a source of evidence, and Ontario's Health Planning Toolkit, which identifies expert opinion as a type of data [23,33].

A handful of studies specifically focused on tacit knowledge have been carried out in the clinical health domain. Tacit knowledge has been shown to aid in the interpretation of explicit knowledge taking the form of standardized outcome measures [34], or to complement technical expertise during the delivery of healthcare [35]. Researchers have also shown that clinical practitioners draw on tacit knowledge to address health problems [36,37]. To illustrate, Herbig *et al. *[36] studied the responses of nurses to hypothetical emergency situations. They found that while similar levels of explicit knowledge were used by the nurses who successfully accomplished the emergency task compared to those who were not successful, there was a marked difference in the levels of tacit knowledge employed. Thornton [38] notes that while guidelines can inform practitioner decision-making, these explicit forms of knowledge are based on a foundation of tacit knowledge and know-how.

Other studies have reported the importance of tacit knowledge for optimal team-based clinical practice [6,34,39]. For example, Gabbay and le May [6] discovered the negotiated and co-constructed nature of knowledge in their study of nurses' and general practitioners' collective decision-making. Study participants used collectively reinforced tacit guidelines based on experiences and interactions in fluid communities of practice rather than drawing on research findings or explicit practice guidelines. Gabbay and le May suggest that discussions were important for sharing, testing, and internalizing these collective 'mindlines.' Studies that focused on team-held tacit knowledge point to the importance of interactions and discussion for joint sense-making.

One difference between the management and clinical perspectives is worth noting. As expected, the management perspective was mostly oriented around the knowledge that an organization held, and individual workers were seen as contributing to the goal of organizational-level competitiveness and advantage through organizational learning. Consequently, strategies to capture tacit knowledge were linked to standardized, organizational-level knowledge management processes and structures. In contrast, the clinical perspective demonstrated a strong interest in the individual worker (nurse, physician) or the team, where tacit knowledge was thought to be associated with experience. The purpose of identifying and capturing tacit knowledge in this context was primarily to contribute to practice performance and training. Organizational benefits were secondary. These two perspectives were sometimes reflected in the terms 'knowledge' (in management) and 'knowing' (in practice). Nevertheless, scholars in both fields continue to ask how tacit knowledge can transform and sustain a competitive organization or professional practice; few, if any, studies make the link between tacit knowledge and outcomes [13].

A number of studies have focused on methodologies or techniques for eliciting tacit knowledge. Some researchers, such as Pavitt [40] and Sobol and Lei [41] have advocated for direct observation, interaction, and discussion in order for researchers to uncover the tacit knowledge that participants hold. Others, such as Sternberg and Wagner [42], have taken a storytelling approach, with the assumption that tacit knowledge is revealed through narratives. Nonaka [43] describes the process of making tacit knowledge explicit as having three phases: the first is in the use of metaphors to verbalize tacit knowledge, the second is linking metaphors to larger analogies, and the final stage involves creating a formalized model. Another methodology that has been used is the critical decision interview method, which asks participants to describe a critical situation that happened recently and then probes for situational and behavioural information that reveals tacit knowledge implicit in the participant's decisions [44,45]. A more proactive model can be found in the theoretical complex clinical scenario, which asks a health practitioner to make decisions about a challenging theoretical clinical situation and elicits tacit knowledge from responses [46]. Some highly specified approaches include using a knowledge acquisition technique called the Ripple Down Rules (RDR) [47] or a linguistically-based approach called the Grammar-targeted Interview Method (GIM) [48] to draw out tacit knowledge. One framework that combines a number of concepts used in the techniques above is Ambrosini and Bowman's methodology [11], which uses semi-structured interviews (allowing narratives and metaphors to develop), causal mapping, and observation techniques. We adopted this approach and supported interactions among participants through focus groups (see Methods section). This study reports on the ways in which tacit knowledge is used in a public health setting to contribute to program planning.

## Methods

### Design

The study research question was: how do health practitioners apply tacit knowledge in public health program planning? Program planning was defined as the one-time effort of planning, tied to a specific initiative, which preceded program implementation (as opposed to ongoing program planning efforts). Types of public health initiatives that would be put forward include such things as community capacity building strategies; the promotion of tobacco prevention, physical activity or healthy eating strategies with specific populations; or campaigns to encourage attendance at disease prevention screening clinics.

Qualitative research is used to understand deeply a complex phenomenon by collecting detailed data based on individual, and sometimes collective, experiences and understandings. This study was designed as a qualitative narrative inquiry, which is based on the assumption that humans make sense of the world by telling stories, and human action is portrayed in these stories [49,50]. In this way, stories are constructive as well as reflective. Narrative inquiry was used to uncover the details of program planning that were significant to those who have lived those events, and provide insight as to the explanatory frameworks drawn upon to make sense of why events occurred as they did [51]. As Riessman and Quinney [52] point out, there are two key components of narratives that distinguish them from other types of qualitative approaches: sequence and consequence. Sequence refers to the order in which the steps and events related to planning are described in a narrative. The study participants choose which parts of an experience to highlight, what to describe, and what to leave out of the telling. Thus, a plot is constructed and situated both spatially and temporally. Events are connected in a particular order through the telling of a story so that a particular outcome or consequence is emphasized. As such, the individual and collective narratives of team planning collected in this study are viewed as participants' attempts to both make sense of how they engaged in planning for a specific initiative and also to convey the key aspects of their planning process as it occurs within their contexts.

### Study setting and participants

Ontario, Canada is a large geographic area with a population of 13.2 million people [53]. Public health services are administered in the areas of environmental health, emergency preparedness, infectious disease prevention and control, family health, and chronic disease and injury prevention. These programs are delivered through 36 public health units in the province, each of which is governed by a board of health and administered through a medical officer of health. All medical officers of health are in turn responsible to the Chief Medical Officer of Health (CMOH) for the province; the CMOH reports directly to the Ontario Minister of Health and Long Term Care. The annual budget for public health in Ontario has been steadily increasing over the past decade, reaching $783.9 million for the 2010-2011 fiscal year [54]. Of the 36 public health units in Ontario, any units that were already involved in KT research or that were part of our previous pilot study were ineligible to participate in this study. Four public health units were purposively selected for maximum variation, based on geography and academic affiliation, from the remaining list. Fourteen health units declined to participate, most often due to heavy workloads.

Directors of Chronic Diseases (or their equivalents) from each of the four health units provided organizational-level consent for the unit to participate in the research. Directors were also asked to identify which teams had been involved in planning chronic disease prevention interventions in the previous six months. The Health Sciences Research Ethics Board of the University of Western Ontario approved this study.

### Data collection and analysis

**Figure 1:**
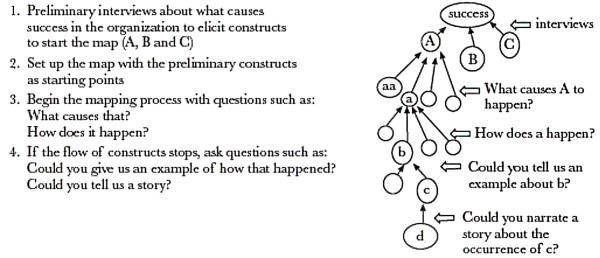
**Illustration of Ambrosini and Bowman's framework for eliciting tacit knowledge**. Figure of Ambrosini and Bowman's framework for eliciting tacit knowledge with detailed description of four key steps in the process. Black Publishers Ltd., reprinted with permission.

We adopted a methods framework outlined by Ambrosini and Bowman [11], developed for the specific purpose of eliciting tacit knowledge. This framework consisted of multiple steps, beginning with individual interviews that ask questions intended to draw out constructs, which are then used as the basis for causal mapping. These interviews could either follow a self-Q format (in which the interviewee asks and answers their own questions) or a semi-structured format. In the interviewing process, attention should be paid to the use of metaphors, as the authors argue that they can be used to express tacit knowledge. Once constructs are identified from the interviews, they are used in the collective mapping process. Questions are asked about the constructs such as 'How does this happen?' or 'What causes this to happen?' that elicit tacit knowledge. Please refer to Figure 1 for an illustration of the mapping process. Beyond interviews and causal mapping, Ambrosini and Bowman state participant observation as a complementary technique for uncovering tacit knowledge, but acknowledge that time constraints may make that difficult.

In our process, we used two key steps from Ambrosini and Bowman's framework. The first step included individual semi-structured interviews designed to elicit participants' narratives about the planning initiative. The second phase involved focus group discussions in which participants drew collective causal maps about a planning initiative [55]. (Note: the maps were used as a tool to facilitate focus group discussion, not as a source of data).

#### Semi-structured interviews

As per the Ambrosini and Bowman framework, individual, semi-structured interviews were used to understand individuals' stories of how planning teams access, make sense of, and use various types of knowledge. Interviews lasted approximately 45 minutes and took place within the participants' workplace. The interviews (n = 24) were recorded and transcribed verbatim. The interviews were designed to elicit stories; specifically, participants, composed of managers and front-line practitioners, were asked to tell a story about recent experiences in program planning.

Narratives were then constructed from the interview transcripts. This process involved reading through the transcripts, re-ordering the main story lines using a chronological framework, and then re-inserting transcript material into the chronological framework [56]. The research team then came together to look for commonly held narratives across all participants and to identify the key constructs described by participants [57]. The narratives served two purposes. First, they allowed the identification of nine specific planning initiatives for the subsequent focus group stage of research; we invited team members associated with these planning initiatives to participate in the focus groups and we used the planning initiative as the 'particular instance' to anchor the discussion. Second, the narratives revealed common constructs with which to organize analysis of the focus group data. These constructs related to the sequence of events in the planning initiatives and were: discovering the opportunity, bringing together the planning team, gaining commitment, and working out program details.

#### Focus groups

Members of each planning team participated in a focus group lasting approximately 1.5 hours; those who participated in the previous interviews were joined by their team colleagues. Teams were asked to think about the recent program they were involved in planning (the particular instance) and to describe all the factors that contributed to the planning. Teams were then asked collectively to construct a diagram or causal map depicting all the factors involved in program planning. According to Ambrosini and Bowman [11], causal maps are representations of individuals' experience of reality that emphasize the linkages between events. These maps are a means of eliciting tacit knowledge because they focus on action and skills. The maps were a collective representation and were co-constructed as a group activity. Having the team come together to describe the planning process also enhanced the comprehensiveness of the story because multiple perspectives were included and negotiated.

The development of the causal map started with a broad question (*i.e*., Tell me about the initiative that you recently planned and how that happened?). The moderator then asked questions (*e.g*., How does that happen? What causes that? Who is involved? What influences that?) in order to uncover the underlying tacit and explicit knowledge informing program decisions. Probes were used to elicit specific information about how tacit knowledge was used. For example, participants were asked to explain what information they relied on for key decisions. Focus group sessions were recorded and transcribed.

Transcripts for the focus groups were analyzed at two levels (AK, DR, and SS). First, transcripts were coded into the stages of planning constructs that emerged from the semi-structured interviews: discovering the opportunity, bringing together the planning team, gaining commitment, and working out program details. Second, within each of these planning constructs, data were analyzed thematically for tacit and explicit knowledge, i.e., patterns in the data were encoded and then interpreted thematically after detailed readings of the transcripts [58]. In particular, the analysis gave prominence to the use of metaphors in the discussions because metaphors help express what is difficult to articulate and are a way of capturing tacit knowledge [59,60]. Other cues for tacit knowledge included the use of 'we (were, did, planned),' and the use of the phrase 'we know...' or a variant of the word 'intuitive,' indicating collectively-held implicit knowledge. An additional file shows the construct clustering and development process more thoroughly [see Additional file 1].

Multiple strategies were used to support a rigorous process. During the focus group, a guide was used to generate a discussion focused on the particular program while still allowing the participants' perspectives and experiences to emerge. Although it is recommended that focus groups be composed of six to eight participants [61] we knew, *a priori*, that some teams had fewer members. We felt, however, that the teams' common involvement in the program of interest precluded joining different teams simply to form a bigger group. In other words, we kept intact, small teams in order to motivate rich conversations that would promote comfort and honest responses. Digitally recording the discussion ensured accurate description [62]. Participants were also sent the de-identified transcripts, and were invited to clarify the meaning of any text segment in the transcript; corrections/additions were noted. We were aware that the moderators (either AK or SS) had some familiarity with the program under discussion on account of the narrative analysis; to minimize imposing our views on the groups' discussion, we made a concerted effort to keep probes as general as possible and to refrain from referring back to the narrative findings. Further, threats to interpretation were reduced by having multiple researchers read and analyze the data independently and then come together for team analysis, followed by a larger team meeting where team members not involved in the analysis, and coming from diverse disciplinary perspectives, could ensure that comments were being interpreted reasonably [62].

## Results

Four public heath units (in Sites A to D) agreed to participate in this study. Site A is a large city of about 100,000 residents that depends on natural resources as key economic industries. Site B is home to more than 500,000 people and is one of Canada's most industrialized cities. Site C is a county composed of a few cities and several municipalities, with a total population of about 100,000. Its economy is driven by the automotive industry and agriculture. Site D is comprised of several municipalities and is home to approximately 50,000 people. While it has a strong industrial sector, its economy is still largely agricultural-based.

In all cases, more than one team within a health unit was interested in participating in the study, as indicated in Table 1 by the column that lists number of semi-structured interviews conducted at each public health unit, as well as the number of focus groups per site. All (but one) individuals who participated in the narrative interviews also participated in the focus groups. From the descriptive data that were gathered from 21 informants, it was found that the majority were female (19/21, 90.5%) and over 40 years of age (14/21, 66.6%). By and large, the informants had nursing as a disciplinary background (15/21, 71.4%), although some had a background in nutrition (3/21, 14.3%). While over one-half of the informants had worked in public health for under 10 years (12/21, 57.1%), it was also common to find nurses working in public health for 10 to 19 years (4/21, 19.0%) or 20-29 years (4/21, 19.0%).

**Table 1:** Number of individual interviews, focus groups and focus group participants

Site	Total # of Individual Interviews	Total # of Focus Groups/Teams	Total # of Focus Group Participants
A	6	A1	2
		
		A2	4

B	4	B1	3
		
		B2	4
		
		B3	8

C	5	C1	8
		
		C2	4

D	9	D1	6
		
		D2	10

**Totals**	**24**	**9**	**49**

Below, we present the analysis of the focus group data, organized by planning construct, to reveal the different ways in which tacit knowledge was used to plan the public health initiative. The analysis is accompanied by illustrative quotes drawn from the discussion. Sections of quotes consisting of such things as 'um' or 'hmm,' the colloquial use of 'like,' and repeated words have been removed for the sake of clarify; words that have been added to provide context are in square brackets.

### Discovering the opportunity

Discovering the opportunity was related to discussions about how the idea or direction for the new or diverging program originated. This construct is similar to the initial phase recommended by traditional evidence-based, decision-making protocols, which involves identifying a practice-based issue and turning the issue into a searchable practice-based question. Here, rather than focusing on a problem or practice-based issue and then turning to the research literature, tacit knowledge seemed to emerge in multiple ways as program practitioners tried to identify opportunities for programs that 'fit' with the community in question.

Informants described combining explicit and tacit forms of knowledge in order to decide on what direction they would take in program planning. In many instances participants described drawing on explicit information sources (*i.e*., research, conference presentations), although such sources were often complemented by inspiration from tacit knowledge about broader contextual considerations. Synthesizing these sources lead to a sense of a 'buzz' about a particular health topic that, in turn, meant that it was the 'right' time for a particular type of initiative:

'I really take the information [from research] and put it into the bigger picture, to take the pieces, and say okay what can we do with all this information, where can I go from here...' (FG C1)

Participants mentioned that informal networking was important to discover ideas or directions because there was little time available to read electronic sources of information. Hallway conversations at conferences were noted as important venues at which to share experiences of successes or difficulties with particular programmatic approaches, thus drawing upon tacit knowledge acquired through experience.

Some focus group participants described a deliberate search for a local opportunity if a mandate for certain activities or outcomes was initiated by government. This search involved combining tacit knowledge about the community with the provincially mandated requirements for public health:

'I don't think there's a set process. We know what the mandate is, it's healthy eating and stress and smoking, so we come around the table and just kind of say okay what are the gaps... are there particular groups that don't have access, are there particular issues within different communities... the mandate might fit and how might we do it... and we have some discussion...' (FG B3)

### Bringing together the planning team

Participants described knowing, based on previous planning experiences, that the composition of their planning team was a key consideration related to the success of the planning process, in part or perhaps because timelines were often tight. Participants felt a well-connected team (both internally and externally) made planning more efficient and less demanding. They also used their tacit knowledge about their colleagues when deciding who to invite onto the team:

'The other thing that we would have around the table when we take on a project like that is you have some sense of whether or not people [*i.e*., colleagues] around the table will go the extra mile for the project, which is never what it seems,... it's going to be twice as much time and we need to know if people around the table [our colleagues] are going to bail.' (FG B1)

Team composition was also related to the skills and level of trust that were brought to the table because of prior shared work experiences. In other words, when inviting colleagues onto teams, the participants used their tacit knowledge of who the 'right' colleagues were for the planning process:

.'..these are people that I've worked [with] on projects ever since they came to the health department and so when we come to a meeting we have some understanding [of] what I bring to the table, what (name) brings to the table, and so on,.. the trust, so if (name) is coming up with something we can be sure that she has massaged that intensely [has done her homework before providing input]...' (FG B1)

Professional credibility was noted as important for program success in some cases. For example, nutrition-related health messages that were supported by dietitians were seen to be more compelling than if supported by other public health practitioners.

One focus group discussed an implicit framework that the team worked from, highlighting team-held tacit knowledge connected to the composition of the team:

'One of the things that we actually had was a model, we didn't have a specific model, but I mean in our mind, it was women's empowerment to increase their ability to take care of their own health and that was sort of at the base of the whole thing...' (FG B2)

### Gaining commitment

Gaining commitment included such things as securing funding, and/or establishing or utilizing partnerships to initiate the program. For example, the quote below illustrates the intuitive challenge of selling a new idea to a community. It also highlights the idea that 'knowing' that things take time is important knowledge, which is developed through experience:

'That you have so many commitments and connections with the community and that the larger relationships are really good, that there's a certain expectation as to what you should be doing so it takes a long time to make a shift here and then make a shift in the community. And some of that is knowing that change takes time. So I'm not discounting that but I think we need to respect that.' (FG D2)

Tacit knowledge was also attributed to ways of working with community partners in order to secure a platform to launch a program:

.'..but we're in partnership with the [organization], with the [organization], with the [organization]. It's a group of people who all recognize that there is a program that needs to be delivered in the community but it's not any one of us that's really taking the lead, we recognize there is a benefit to working together on these things.' (FG C2)

However, in general, the gaining commitment stage of program planning seemed to invoke fewer examples of tacit knowledge than the other stages.

### Working out program details

Teams often described a taken-for-granted process in which they engaged to plan a program. These operational details reflect tacit knowledge regarding the aspects of the program that must be worked through, including things like identifying the target group, engaging them in the program, using partnerships to implement the program, and tailoring the program. Some participants described how they worked with the community to make programs more acceptable, and the way in which they did this seemed to be based on tacit knowledge related to community needs, and how to best connect and communicate. In the quote below, the respondent describes using tacit knowledge to identify how to communicate the health message instead of describing what the research literature recommends:

.'..and the other thing with the [teams'/nurses'] intuitive piece is we're all, or most of us, are from rural areas and in the rural areas it's the human connection, it's the hair stylist in the home and Sally from down the street coming to get their hair cut, and it's that kind of communication that has a lot of power, I think, in influencing people's decisions.' (FG D1)

In theory, program development is based on a needs assessment of community health issues. However, public health teams mentioned deliberate needs assessments to focus program details only a few times. It was noted that new team members (especially new graduates) brought explicit knowledge in the form of 'book smarts' (*i.e*., recent training). Sometimes participants relied on stories told to them by the peer educators or community advisors working with the health unit, thus collectively building tacit knowledge of 'the community,' or a sense of the 'pulse,' through discussion:

.'..because we know all the players, things like needs assessments, stakeholder analysis, you know it's hard to stop and do it formally when it's always current [*i.e*., our knowledge], we always sort of have our finger on the pulse sort of thing...' (FG A2)

Informants also used the integration of tacit knowledge with explicit knowledge to understand a community health problem, as illustrated by the following quotation:

.'..as a public health nurse,... we do a lot of different things, it's like a mile wide and an inch deep. And so we have our hands in lots of pots... and I suppose there are lots of influences that come over that [long] period of time through my work and my exposure in the community, my personal life, and through work. Of course you're looking at information, the demographic information, the epidemiological information, you know disease trends in our community, we know that diabetes is very high here, we know that, that the cardiovascular diseases is really prevalent and you don't have to know anything and still see that obesity is a huge problem in our communities as well.... I think all of those things just sort of culminated...' (FG A1)

There was also a tacit recognition of balancing explicit knowledge with partnering expectations:

'We run this interesting line trying to figure out how much public relations stuff you do and partnership supporting that you do largely on public donations, and how much we can do with evidence-based [programming], because our partners often don't share the same view-point when it comes to evidence, they don't have to care about it so they don't want to care... so you do it because you don't want to lose them as a partner and you know they'll walk if we tow a real hard line.' (FG D2)

### Awareness of tacit knowledge

Focus group participants demonstrated their awareness of tacit knowledge and provided clear examples of this a few times:

.'..so she was away for the year... and she had to put together a review of the briefing notes, so we gave her all the briefing notes that we had done and then she's trying to put that together in her head, and they've been very detailed, she came around and she had to ask both of us what were the nuances of the details, right, so how did that happen? We necessarily document that in writing, but we know the nuance... And that was important for her to know...' (FG B1)

Focus Group Moderator: 'so talking about that intuitiveness is that what everyone felt, like this should be a good thing or what exactly?'

Participant: '... and so it developed over the years, you develop ways of, you develop this 'I think this will work' because we've seen where it does...' (FG D1)

One focus group described using tacit knowledge to evaluate explicit knowledge, demonstrating the dialectic nature of both types of knowledge:

'Also, it takes time to realize that just because it's [that breast-self-examination was ineffective] in the paper doesn't mean it's true. The Cancer Society was all in the paper, it was all very controversial, and some people took it at face value just because it had a good foundation name with it.' (FG D2)

## Discussion

The findings of this study demonstrate that tacit knowledge is drawn upon, and embedded within, various stages of the program planning process as described by our sample of public health teams. Participants did not often explicitly state the use or awareness of tacit knowledge. That is, in most instances they did not come out and say, for example, 'We used our tacit knowledge about the possible solutions to the childhood obesity problem to define the parameters of this program.' Rather, through our careful analysis of the focus group discussions across nine focus groups, as described in the methods and as illustrated by the quotes provided, it was clear that tacit knowledge played a strong role in program planning. The virtual absence of tacit knowledge exploration in the public health literature is a significant gap limiting current understanding of KT in this field. An exception is Yoshioka-Maeda *et al. *[37], who explored the tacit knowledge used by public health nurses. Their findings pointed to the importance of tacit knowledge for identifying community problems, and then being able to respond quickly with needs-based programs. Our findings extend beyond needs assessments to illustrate the role of tacit knowledge during the continuum of program planning. In particular, almost routine ways of conducting the planning process, akin to tacit skills or practices, were articulated. Analysis of the data also revealed how tacit knowledge was drawn upon to attend to the specifics of the particular initiative being planned, conforming with previous research studies [6,34]. Our study pointed to the importance of tacit knowledge for understanding and anticipating how team members will function, as did Friedman and Bernells' research [39] on healthcare teams and tacit knowledge.

Theoretically, these findings support the utility of models that consider both tacit and explicit knowledge in a dynamic interchange, such as that of Nonaka and Toyama [14,18] for the public health context. The data reflected the importance of previous social interactions, and how tacit knowledge was created through shared experiences, such as hallway conversations at conferences, or having worked previously with community stakeholders. The findings described interplay between tacit and explicit knowledge. The data also pointed to the challenges in attempting to externalize this tacit knowledge, *e.g*., needing to ask for the 'real' story, despite getting briefing notes.

Methodologically, we found that Ambrosini and Bowman's framework [11] was able to elicit articulations of tacit knowledge, particularly in focus groups where team members articulated the knowledge informing key events in the program planning process. Like Gabbay and le May's [6] study that identified collective 'mindlines,' in this study previous social interactions facilitated the creation of individual knowledge, and then, in the context of the focus group, the generation of causal maps provided the opportunity for joint sense-making for this research study. We agree with other researchers who suggest that tacit knowledge may not ever be completely revealed, and expect this to be the case with the findings presented here [11,14]. With this in mind, a qualitative narrative approach--asking public health practitioners to describe the situations where they used tacit knowledge and to share their stories about this--would be more likely to uncover instances of tacit knowledge use than would a close-ended instrument, such as that developed by Leonard and Insch [9]. Telling the story might have even elicited dimensions of tacit knowledge of which the respondent was unaware, given the taken-for-granted nature of particular practices.

This study aimed to improve our understanding of how tacit knowledge is used to inform program initiatives in public health. There were several reasons for this focus. First, explicit knowledge is not always available in public health to guide program planning. Second, in some areas explicit knowledge is available but is not used. Third, explicit knowledge may not take into consideration the local context in which public health units are situated. Fourth, the practitioners who carry out public health program planning and implementation are experts in their fields. They described tapping into the tacit knowledge and expertise they had accumulated through years of practice. This included their own tacit knowledge, as well as that of their team members and community partners. While this tacit knowledge may be important for the success of program planning and implementation, these expert resources are currently under-represented in traditional evidence-based discourse. Thus, this study supports the assumption that tacit knowledge is an essential feature in public health that requires further exploration.

The importance of eliciting tacit knowledge is tied to the evidence-based medicine movement, which has given primacy to the use of research findings by practitioners without due attention to the role of other sources of knowledge [63]. This study demonstrates how tacit knowledge is taken into account by public health practitioners. The findings may also contribute to making sense of evidence-based, decision-making models with components that include research evidence, setting/circumstances, patient preferences, and clinical expertise (*e.g*., see [64]), where tacit knowledge may be woven into our understanding of clinical expertise. From another perspective, Chen [65], in his model of integrative validity, puts forth a strong argument for assessing real-world viability, *e.g*., inviting stakeholders' tacit knowledge about the practicality, suitability and acceptability of a public health program, before turning to issues of program efficacy or effectiveness [65].

The evidence-based medicine movement has taught practitioners that explicit knowledge, such as research literature, demands critical appraisal before utilization. There are internationally accepted standards for appraising research studies, *e.g*., CONSORT (Consolidated Standards of Reporting Trials Statement) and AMSTAR (Assessment of Multiple Systematic Reviews). While we have positioned tacit knowledge as a desirable feature, it is recognized that not all tacit knowledge is 'good' tacit knowledge. All knowledge, regardless of its explicit or tacit nature, demands critical appraisal. It is likely that some types of tacit knowledge might in fact lead to ineffective practice. Nonaka and Toyama hint at this when they remark that 'high-quality tacit knowledge is the source of sustainable competitive advantage...' [18], but at this stage the difference between high- and low-quality tacit knowledge is unclear, nor do we understand how conflicting tacit knowledge plays out in decision- making situations.

That some tacit knowledge might be associated with ineffective practice suggests a negative side to tacit knowledge. It has been suggested that entrenched tacit knowledge can prevent effective adaptation to environmental changes, such as legislative requirements [11]. In those cases where tacit knowledge is deeply entrenched in practice, this might serve as a barrier to accepting new research that conflicts with tacit knowledge, which might result in harmful outcomes. Tacit knowledge might also act to stall knowledge creation rather than promote it [34]. While this study did not deliberately examine the negative implications of tacit knowledge, we mention these issues because they are important aspects to consider in future studies of 'know-how.'

Furthermore, literature about tacit knowledge stresses its context-dependent nature, and context is both personal and organizational. While explicit research knowledge is related to methodological rigor, tacit knowledge is related to relevance or real-world viability [65]. In fact, in our study the degree of influence of tacit knowledge appeared to outweigh knowledge available through explicit means. The dilemma then is how an organization can identify and support contextually-bounded tacit knowledge that leads to effective practice. Equally perplexing is how an organization then can integrate tacit and explicit knowledge more fully, resulting in more effective practice.

The findings ought to be considered in light of the study limitations. The methods used to uncover tacit knowledge follow suggestions from the literature to support interactions, discussions, story-telling, focus on a particular instance and group mapping. We were unable, however, to carry out participant observation, which might have revealed a skill-based aspect of tacit knowledge not discussed by respondents. Consequently, the findings might under-represent the level of tacit knowledge used in public health program planning. The methods involved inviting those who revealed individual narratives about program planning to also participate in the focus groups where a more comprehensive, group-based account of program planning was elicited. We suggest that participating in the first phase did not significantly influence the outcomes of the focus groups on the grounds that more detailed probes with a focus on a particular instance were used in the focus groups, in contrast to encouraging broad, uninterrupted story-telling with minimal questioning in the first phase. Further, over one-half of the focus group participants were comprised of new individuals. Future research might systematically investigate this methodological question. We have claimed that the focus groups provided the opportunity for joint sense-making of tacit knowledge in the same way that previous researchers have shown that social interactions provide a forum for co-creating shared stories. This claim could have been better substantiated if we had assessed effects of the focus group on participants, or determined if tacit knowledge became easier to access over time and joint sense-making more readily observable. An interesting question for further study is whether the important data collection element was the focus group, the map-creation or both, and whether joint-sense making is useful for future program planning. Finally, we remind readers about the knowledge claims that can be made from this narrative inquiry, for which the goal is not to generalize findings nor capture all of tacit knowledge. This type of study seeks to raise insights and show a phenomenon in action. In our case, we may know that practitioners use more than literature, but the interest was in understanding how that happens, how it is described and what insights can be learned about this that might contribute to training or team development.

## Conclusion

The results of this study provide insights into how tacit knowledge comes into play during the program planning process in public health. These results will be useful in guiding the development of future KT strategies for public health organizations and decision makers. For example, it is clear from these results that in order for KT strategies to be successful, careful attention to the importance and influence of tacit knowledge in program planning will be needed. In addition, strategies will need to be developed that model how explicit and tacit knowledge can be used together in order to arrive at planning decisions that integrate the best knowledge about what works in public health, along with considerations of how to adapt explicit knowledge to suit different contexts. These results also suggest that KT strategies will need to focus on changing the culture of public health toward one that values the integration of explicit and implicit knowledge, as well as the development of knowledge, capacity and skills on how to integrate these two forms of knowledge successfully.

## Competing interests

The authors declare that they have no competing interests.

## Authors' contributions

AK and NE designed the study. All authors contributed to the execution of the study. DR provided the methodological expertise for data analysis, and all authors contributed to interpreting findings. AK wrote the first draft of the manuscript, with assistance from SS. All authors provided critical feedback on earlier versions of the paper. All authors read and approved the final manuscript.

## Supplementary Material

Additional file 1 **Construct Clustering and Development**. A series of tables showing how constructs were developed and clustered into their final categories. Click here for file
