# Supplementary material for: The use of tacit and explicit knowledge in public health: a qualitative study
Source: Implement Sci. 2012 Mar 20;7:20. doi: 10.1186/1748-5908-7-20 (PMC3325865; doi:10.1186/1748-5908-7-20)
Supplement: Additional file 1 — Construct Clustering and Development. A series of tables showing how constructs were developed and clustered into their final categories. [file 1748-5908-7-20-S1.PDF]

## Additional File 1: Construct Clustering and Development

### Table 1-a New Construct Name: DISCOVERING THE OPPORTUNITY

| **Emerging Constructs from Individual Narratives Identified by Research Analysts** | **Description** |
|---|---|
| Finding the buzz | The term "buzz" came out of the team; What is going on at large/the bigger picture; Where does program fit within priorities? Buzz appears to represent the interest levels in a particular area. It can be flowing into or out of the program/service area. Can be generated by new research knowledge that sets program focus in a new direction. Media buzz and professional buzz are identified as different considerations |
| Unearthing the Knowledge | Who has the knowledge and how is it accessed? Knowledge is exchanged through many sources and at different levels; It is a complex interaction of program planners, delivery agents, community, media, etc that influence program direction and success; Competencies plus tacit knowledge impact on success of an endeavour |
| Utilizing the literature | |
| Getting the idea | Where does the idea for the program come from? |
| Finding the Buzz | Ideas coming from: mandate, other successful health units/organizations, research findings, community needs. Seeing opportunity from: available funding, research findings, trends, and community support. |
| Assessing What's Available | ID skills, resources, activities to guide what is needed in terms of level of direction and resources to operationalize a new program |
| Carpe Diem vs. Slow and Steady | Making a choice between an arising opportunity and continuing with an existing program |
| Finding the right fit | Identifying opportunities for programs that "fit" with the community in question; finding partners that "fit" with program objectives (First half could fit under discovering buzz, second half under establishing program foundation) |
| Identifying a need | |
| Carpe Diem | Seizing opportunities as they arise |
| Building on What Exists | IDing opportunities to link new and old programs, projects, extend what exists with what is emergent |
| Bringing It - experience to the Banquet Table | Sorting through what are the real opportunities/challenges as opposed to what is fluff – knowing what is relevant to the task/community (experiential) |
| Utilizing/Evaluating Explicit Knowledge | The act of seeking out and assessing research evidence to build the foundation of the program |
| Developing a Program Rationale | Finding the evidence to support program plans and directions to justify directions, meet ministry guidelines, etc (partners do not have to justify their actions in this way) |

## Table 1-b New Construct Name: BRINGING TOGETHER THE PLANNING TEAM

| **Emerging Constructs from Research Analysts** | **Description** |
|---|---|
| Making the Commitment | Hard work, who will commit? Does this link to team forming? |
| Forming a Team | Aggregated data about the team coming together; New approaches and ideas stem from newest members? Experience brings tacit knowledge about who can contribute to a working group and how; know who to approach and how |
| Multiple Memberships | |
| Forming the team | Collecting people to form a team; collecting people based on goodness of fit (including knowledge they bring, availability and support of objective). |
| Conditioning Professional Support | Knowing who does what within the health unit and who will support is necessary for good program development. Linking in strengthens and grows the overall HU objectives. |

## Table 1-c New Construct Name: GAINING COMMITMENT

| **Emerging Constructs from Research Analysts** | **Description** |
|---|---|
| Finding the Support | Board; Funders (previous not successful or long term) (tacit experiential what works/what doesn't); Searching for a secure platform to launch a program; The support is critical to a programs success in this case there were many community members, staff and management who provides different types and levels of support |
| Financing/sustaining programs | |
| Getting resources | This can be monetary (funding) or other resources such as brochures/posters. This does NOT include human resources |
| Getting support | This can be internal or external; can include people, media, organizations |
| Finding support | Finding support in-house, from the community, from higher-level groups (e.g. government), financial support. |
| Conditioning Professional Support | Knowing who does what within the health unit and who will support is necessary for good program development. Linking in strengthens and grows the overall HU objectives. |
| Accessing Grants | Finding funding for programs increases credibility and commitment from stakeholders |
| Making program objectives & outcomes public/ Information sharing | Making objectives and plans transparent, making outcomes public (public awareness for public approval), sharing planning information and research with other health units and organizations. |
| Spreading the word and Building Awareness | Communicating with the community to create awareness of programs and services |

## Table 1-d New Construct Name: PARTNERING AND ENGAGING

| Emerging Constructs from Research Analysts | Description |
|---|---|
| Making program objectives & outcomes public/ Information sharing | Making objectives and plans transparent, making outcomes public (public awareness for public approval), sharing planning information and research with other health units and organizations. |
| Spreading the word and Building Awareness | Communicating with the community to create awareness of programs and services |
| Keeping Connected | Sharing with network; Getting feedback and hearing what works/doesn't work from others; Feeding the buzz; Sustaining relationships, makes the start-up and delivery of new or altered programs easier; Tacit knowledge of community partners and who will support and endeavour and in what way; Give and take provides a joint benefit for HU and stakeholder |
| Partnering/Using Relationships/Engaging the Community | Bring together data around community partners; Examples of community engagement |
| Linking with partners | This can be internal or external; can be community or organizational (other health units) |
| Engaging community/making connections/keeping connections | Communicating ideas with the community, appealing to the community for support (showing how the program will benefit them), taking into account what is best for the community based on community input, maintaining community support through positive connection and interaction. |
| Linking in With Partners | Identifying multiple benefits with partners leads to a sense of shared ownership = shared incentive and shared commitment |
| Connecting with Partners | Raising awareness of all the resources available through various programs to improve outcomes for clients = enhanced referrals, access, etc |
| Forming Partnerships | A work intensive process where different groups/agencies put ideas on the table and talk to service recipients to form program links, supports and ID needs |
| Building Relationships | Goal oriented approach for program development where all partners should be equal in the process of development and execution |
| Partnering Within and Beyond the HU Boundaries | Communicating with NGOs and other agencies with similar interests/objectives strengthens programs |
| Finding the right fit | Identifying opportunities for programs that "fit" with the community in question; finding partners that "fit" with program objectives |
| Connecting with partners | Identifying those in the community with similar interest and connecting with them to further a program plan |

## Table 1-e New Construct Name: WORKING OUT PROGRAM DETAILS

| Emerging Constructs from Research Analysts | Description |
|---|---|
| Pinpointing the key problem | Does knowledge exist? Eliminate the non-issues/what is not the problem; Solution fits the problem; Context specific; How they developed the plan to stay the course |

| | |
|---|---|
| Finding the Focus/Finding the Target | At what time does the tacit knowledge prevent an exploration of new approaches; Does tacit knowledge still hold value where community itself is in transition and this is not recognized (i.e. demographics, primary caregiver in home is changing, family make-up and dynamics) |
| Program Details | Working out the details of the program; where, when, who |
| Finding a target | Deciding on specific objectives and guidelines. |
| Having a clear goal/objective | In a multiple group endeavour, clearly articulating desired outcomes that meet all objectives |
| Meeting the needs | |
| Developing the Program | Developing the program is identified as a sequential process of brainstorming, reviewing evidence, sharing information as the second step |
| Id an overarching strategy | When multiple partners are involved, it is important to id at the start an overarching goal and strategy that is agreed upon to move forward effectively |

## Table 1-f New Construct Name: IDENTIFYING STRENGTHS AND WEAKNESSES

| Emerging Constructs from Research Analysts | Description |
|---|---|
| Contextualizing | Making sure the program will work for the chosen target/community or PH issue |
| Identifying & overcoming barriers | Identifying where changes could be made, making efforts to improve program, learning from past mistakes, learning from programs of other health units/organizations. |
| Meeting The Northern Challenge | Factors effecting program delivery in northern communities that challenge program delivery |
| Ground-Truthing and Visualization | Look at big picture compare strengths and weaknesses of various models extrapolate and incorporate into program design |
| Maintain Philosophical Integrity | Linking with other groups may impose new objectives/approaches contrary to the original philosophy |
| Anxiety versus Tension between partners | Noted as a relevant difference in program connections - anxiety about developing program versus tension existing between partners - impact on program development |

## Table 1-g New Construct Name: EVALUATION

| Emerging Constructs from Research Analysts | Description |
|---|---|
| Piggy backing on existing programs/ Piloting new with the old | Using existing programs to meet new needs and identifying how they fit together and can benefit from one another. |
| Piecing the Puzzle | Making sense of various experiences in order to address concerns and identify issues - taking the small parts to create a bigger picture of an issue |
| Evaluating the program | Steps that are taken to evaluate what has been done both in planning and in implementation |
| Finalizing/Follow-up | Once the program is done, what happens |

**Note that of these seven new constructs, the results from "Partnering and Engaging" are being reported another publication, and the constructs "Identifying Strengths and Weaknesses" and "Evaluation" were subsequently clustered with "Working out Program Details.**
